# Piezo Channels: Awesome Mechanosensitive Structures in Cellular Mechanotransduction and Their Role in Bone

**DOI:** 10.3390/ijms22126429

**Published:** 2021-06-16

**Authors:** Xia Xu, Shuyu Liu, Hua Liu, Kang Ru, Yunxian Jia, Zixiang Wu, Shujing Liang, Zarnaz Khan, Zhihao Chen, Airong Qian, Lifang Hu

**Affiliations:** 1 Lab for Bone Metabolism, Key Lab for Space Biosciences and Biotechnology, School of Life Sciences, Northwestern Polytechnical University, Xi’an 710072, China; xuxia1036916053@mail.nwpu.edu.cn (X.X.); syliu@mail.nwpu.edu.cn (S.L.); liuhua17835064039@mail.nwpu.edu.cn (H.L.); rukang@mail.nwpu.edu.cn (K.R.); jiayunxian1@nwpu.mail.edu.cn (Y.J.); wuzx@mail.nwpu.edu.cn (Z.W.); liangsj@mail.nwpu.edu.cn (S.L.); zarnazkhan@hotmail.com (Z.K.); chzhh@mail.nwpu.edu.cn (Z.C.); 2 Xi’an Key Laboratory of Special Medicine and Health Engineering, School of Life Sciences, Northwestern Polytechnical University, Xi’an 710072, China; 3 Research Center for Special Medicine and Health Systems Engineering, School of Life Sciences, Northwestern Polytechnical University, Xi’an 710072, China; 4 NPU-UAB Joint Laboratory for Bone Metabolism, School of Life Sciences, Northwestern Polytechnical University, Xi’an 710072, China

**Keywords:** Piezo channels, mechanotransduction, bone cells, bone disease

## Abstract

Piezo channels are mechanosensitive ion channels located in the cell membrane and function as key cellular mechanotransducers for converting mechanical stimuli into electrochemical signals. Emerged as key molecular detectors of mechanical forces, Piezo channels’ functions in bone have attracted more and more attention. Here, we summarize the current knowledge of Piezo channels and review the research advances of Piezo channels’ function in bone by highlighting Piezo1′s role in bone cells, including osteocyte, bone marrow mesenchymal stem cell (BM-MSC), osteoblast, osteoclast, and chondrocyte. Moreover, the role of Piezo channels in bone diseases is summarized.

## 1. Introduction

Mechanotransduction is an important process for living cells and tissues by which they experience and respond to mechanical stimuli [1]. Bone, one of the main mechanical supportive structures of the body, experiences various mechanical forces (e.g., gravity, locomotion, exercise, and external vibration) over the body’s whole life [2,3,4]. Mesenchymal stem cells (MSCs), osteoblasts, osteocytes, and osteoclasts, which are the main constituent cell types of bone, are stimulated by matrix stiffness, fluid shear stress, tension, and compression [5,6,7,8]. By sensing and responding to mechanical stimuli, bone cells adapt to the mechanical environment and maintain bone homeostasis [6,9,10]. Moreover, chondrocytes, the primary cell type of cartilage that is also involved in bone development, are also stimulated by mechanical stimuli [11]. Therefore, cellular mechanotransduction is crucial for bone development and physiology, and abnormal cellular mechanotransduction leads to various bone diseases, including osteoporosis (OP) and osteoarthritis (OA) [12,13]. Thus, uncovering the mechanotransducers and the underlying mechanism of cellular mechanotransduction is one of the greatest concerns in bone physiology and pathology research.

Mechanically activated ion channels represent a key type of mechanotransducer that effectively convert mechanical stimuli into electrochemical signals, which is important for both physiological and pathological process [14,15,16]. In 2010, the discovery of Piezo proteins (Piezo1 and Piezo2) that form mechanosensitive cation channels opened a new era of mechanotransduction research [17]. The Piezo family of proteins, including Piezo1 and Piezo2, are large membrane proteins that are evolutionarily conserved. Mammalian Piezo proteins consist of 2500–2800 amino acids [18,19,20]. The Piezo channels play crucial roles in numerous physiological and pathological process by functioning as cellular mechanotransducers [17,19,21,22,23]. Under mechanical stimuli, Piezo channels are opened to make cationic ions cross membrane, which promotes cellular mechanotransduction to adapt to the microenvironment [24]. Piezo1 has recently been discovered to be important for vascular mechanotransduction (e.g., blood pressure regulation) [25], urinary osmolarity [23], cartilage mechanotransduction [26], dorsal root ganglion neuron physiology [27], peripheral trigeminal nociception [28], and other physiopathological conditions. Meanwhile, Piezo2 mainly functions in somatosensory neurons physiology, airway stretch, and lung inflation [29,30,31]. More recently, the Piezo channels have shown key roles in mediating the mechanotransduction in bone cells and are involved in bone disease [7,8,22,32,33].

Here, based on the introduction of the members and structure of Piezo channels, we summarize the current advances of Piezo channels and highlight their role in bone, providing perspectives on Piezo channels for consideration in future studies.

## 2. Piezo Channels

### 2.1. The Genes, Members, and Structures of Piezo Channels

#### 2.1.1. The Genes and Members of Piezo Channels

Piezo channels have two members in vertebrates: Piezo1 and Piezo2, which have the property of being activated by pressure [19]. In 2010, Coste et al. screened out the ion channel protein (coding gene: *Fam38A*) that produces the most stable current and the largest response under pressure stimulation [17]. Subsequently, the protein encoded by the *Fam38B* gene was found through sequence homology [17]. *Fam38A* and *Fam38B* are located on human chromosomes 16 and 18, respectively (gene location information was obtained from the NCBI database). Because the Greek word “pίesi” means pressure, *Fam38A* and *Fam38B* were named as Piezo1 and Piezo2, respectively [17]. Human Piezo1 and Piezo2 consist of 2521 amino acids and 2752 amino acids, respectively [34]. Mouse Piezo1 and Piezo2 are composed of 2547 amino acids and 2822 amino acids, respectively [35,36] (Table 1).

#### 2.1.2. Structure

**Figure 1:**
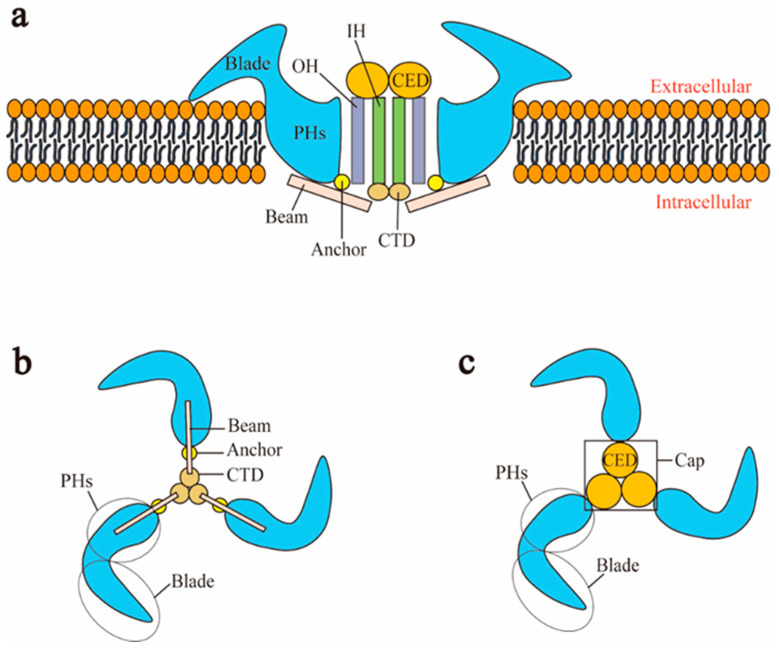
Schematic representation of Piezo1 channel. (**a**) Lateral view; (**b**) bottom view; (**c**) top view. CED: C-terminal extracellular domain; CTD: intracellular C-terminal domain; IH: inner helix; OH: outer helix; PHs: peripheral helices.

Piezo1 and Piezo2 both have a similar homotrimer structure but differ in many aspects. At present, the cryo-electron microscopy structures of mouse Piezo1 and mouse Piezo2 have been obtained [36,37]. However, the structures of human Piezo1 and Piezo2 remain to be resolved. In mouse, Piezo1 is a homotrimer, which is similar to a three-bladed propeller [44]. Piezo1 can be divided into two modules: the peripheral mechanotransduction module and the central ion conduction pore module [44,45] (Figure 1a). The peripheral module consists of the extracellular distal blades, the peripheral helices (PHs), anchors of the transmembrane, and the intracellular beams. The central pore module includes the C-terminal extracellular domains (CEDs), the transmembrane inner helices (IHs) and outer helices (OHs), and the intracellular C-terminal domains (CTDs). The combination of three CEDs forms an extracellular cap [20,24,44] (Figure 1c). The intracellular beam connects the PHs to the CTD [40]. There are many transmembrane helical units (THUs) in the transmembrane region, and THUs are roughly divided into IHs, OHs, and PHs [35] (Figure 1a). The OH is connected to the first peripheral helix near the central axis by a hairpin structure that is formed by four α helices and named the anchor [35]. The C-terminal of each monomer is connected in order as PHs-Anchor-OH-CED-IH-CTD [35,37]. Recently, Geng et al. presented a model of Piezo1′s exquisite plug-and-latch mechanism. More precisely, on the cytoplasmic side, each monomer of Piezo1 has a lateral ion channel: a plug and a latch [46]. Under the pull of the latch, the plug is removed to allow the ions to pass [46]. Zheng et al. found that the intracellular CTDs of Piezo1 can drive the IHs to move by contracting, which rapidly deactivates Piezo1 [47]. These data suggest that the delicate structure of Piezo channels is responsible for its mechanotransduction.

Although Piezo2 is roughly similar to Piezo1 in structure, there are many differences in their details. Cryo-electron microscopy structures of Piezo1 and Piezo2 indicated that the central pore of Piezo1 is dilated, while the transmembrane central pore of Piezo2 is closed [36]. The three constriction sites of Piezo2 at L2743, F2754, and E2757 are apparently dilated in the corresponding positions in Piezo1 at L2469, F2480, and G2483, respectively [36]. The ion-permeating properties of Piezo2 are controlled by E2757, which is the only negatively charged residue in the inner helix [36]. In addition, the diameter, depth, and maximum projected area of Piezo2′s intermediate plane openings are 24 nm, 9 nm, and 250 nm^2^, respectively, while the related parameters of Piezo1 are 18 nm, 9 nm, and 120 nm^2^, respectively [36].

## 3. The Role of Piezo Channels in Cellular Mechanotransduction

The Piezo channels, as mechanical sensors, are responsible for converting the extracellular mechanical stimulation perceived into the biochemical signal generated by its ion entry pore. Then, the electrochemical signal causes a series of intracellular downstream signaling pathways [24,48]. Syeda et al. indicated that lateral tension from lipid molecules activates mechanosensitivity of Piezo1 in vitro [49]. It is speculated that extracellular mechanical stimuli can be converted into tension from lipid molecules in the cell membrane to activate the Piezo channels [49]. The intracellular beam might transmit tension from lipid molecules from the distal blade to the central pore module by means of a lever principle, which opens the central pore for ion-conducting [20,40,44]. Zhao et al. found that Piezo1 uses its key mechanosensitive elements—including L15-16 and L19-20 in the extracellular loop region and L1342 and L1345 amino acid sites in the intracellular beam—to open the ion-conducting pore responsible for ion permeability from a distance in in vitro research [37]. Piezo1 selectively conducts cations, such as Na^+^, K^+^, Ca^2+^ and Mg^2+^, with a slight preference for Ca^2+^ [17]. On the contrary, Piezo2 has the property of non-selective cationic conductance [17].

When Piezo channels are opened/activated, a cellular signaling cascade is triggered by the Ca^2+^ influx through the Piezo channels [24,50,51,52,53]. Zhao et al. considered that the extracellular CED has a lot of negative charge, so extracellular cations such as Ca^2+^ will be aggregated and will flow through the pore of Piezo channels to cross the cell membrane [24]. Piezo1 channels act as shear-stress sensors that promote human and mouse endothelial cell organization and alignment in the direction of flow by promoting Ca^2+^ influx in vitro research [51]. Downstream of this calcium influx in human and mouse endothelial cells, there are protease activation and spatial reorganization of endothelial cells to the polarity of the applied force [51]. By experimentally stretching epithelial cells in vitro, Gudipaty et al. found that mechanical stretch, through the mechanosensitive Piezo1, triggers these cells to pause in early G2 to activate calcium-dependent ERK1/2 phosphorylation and cyclin B transcription, thus prompting these cells to enter mitosis [52]. Exposure to blood flow shear force in vitro activates the Piezo1 channel, with ensuing Piezo1-mediated Ca^2+^ influx in platelets [53]. An increase in intracellular Ca^2+^ level in turn promotes activation of calpain-2 and cleavage of talin1, leading to platelet aggregation [53]. The signaling cascade induced by Ca^2+^ flowing through Piezo channels also takes place in bone cells and chondrocytes [7,26,32].

These data indicate that the Piezo channels play a key role in transforming mechanical signals into biochemical signals in cellular mechanotransduction. Thus, we can present a working model of the Piezo channels. To be specific, extracellular mechanical stimuli sensed by cells, such as fluid shear stress as well as tension and compression forces, causes the lipid molecules on the cell membrane to change so that tension from the lipid molecules is sufficient to activate the Piezo channels [49]. Furthermore, the peripheral mechanotransduction module of the Piezo channels feels the tension from the lipid molecule, opening the central pore module [46]. Because the extracellular CED has a lot of negative charge, the concentration and internal influx of extracellular cations such as Ca^2+^ leads to the activation of downstream signaling pathways [24,50].

## 4. Activators and Inhibitors of Piezo Channels

At present, the activators of Piezo channels have been found to be Yoda1 and Jedi1/2, and the inhibitors of Piezo channels have been found to be ruthenium red, gadolinium, streptomycin, GsMTx4, and FM1-43 [17,19,39,40,41,54] (Table 1).

Yoda1 and Jedi1/2 are known as synthetical activators of Piezo1, while activators of Piezo2 have not been reported. Syeda et al. identified a compound called Yoda1 by conducting high-throughput screening on about 3.25 million low-molecular weight compounds while monitoring the inflow of Ca^2+^ through Piezo1 in in vitro research [39]. Yoda1 optionally opens Piezo1 instead of Piezo2. Yoda1 stabilizes the open conformation of Piezo1 and reduces the mechanical threshold needed for Piezo1 to activate [39]. Wang et al. identified a new set of Piezo1 chemical activators called Jedi [40]. Jedi1/2 act on the upstream blade, while Yoda1 acts at the downstream beam to regulate the activity of the Piezo1 [40].

Ruthenium red, gadolinium, and streptomycin are known to be blockers of many cationic channels, so they can also block Piezo channel-induced currents [17,41]. The peptide GsMTx4 (grammostola spatulata mechanotoxin4) is an inhibitor of cationic mechanosensitive channels [54]. By injecting GsMTx4 in vivo, a polypeptide derived from tarantula venom, into the posterior limb arteries of rats, Copp et al. found that GsMTx4 reduces the rats’ movement pressure reflex due to its effect on the mechanically gated Piezo channels [42]. Drew et al. found that FM1-43, a styrene dye used for fluorescent labeling of cell membranes, could permeate and block mechanosensitive ion channels in dorsal root ganglia neurons in vitro [43]. Eijkelkamp et al. utilized the property of FM1-43 to find that it can block Piezo2-induced currents in vivo and in vitro [55].

## 5. The Role of Piezo Channels in Bone

Bone is a finely mechanosensitive organ and constantly adapts its shape and internal structure to mechanical loads, including body weight, exercise, and gravity [56,57]. Bone homeostasis, maintained by modeling, remodeling, and tissue repair, is regulated by coordinated activities of the bone cells through a process termed cellular mechanotransduction triggered by mechanical stimuli, including fluid shear force, compression, tension, and so on [58,59]. Bone cells include osteoblast lineage (roughly divided into mesenchymal stem cells (MSCs), osteoblasts, and osteocytes) and osteoclasts [60,61]. Osteocytes are terminally differentiated osteoblasts that are derived from MSCs [62,63]. Osteocytes play a vital role in bone homeostasis by regulating the formation and activity of osteoblasts and osteoclasts [63]. In addition, the cartilage that connects the bone in the joints is also an intrinsically mechanosensitive tissue composed of chondrocytes as the only cell type [64]. Chondrocytes, one of the differentiation directions of MSCs, regulate the metabolism of the cartilage extracellular matrix (ECM) to adapt to the mechanical stress environment [65]. At present, many studies have found that Piezo1 is expressed in bone and plays an important mechanotransduction role there [8,32,33,66,67]. Moreover, Piezo channels (Piezo1 and Piezo2) are expressed in chondrocytes and participate in the maintenance of cartilage homeostasis associated with mechanotransduction [22,26,68].

### 5.1. Piezo1 and Osteocytes

Osteocytes are the most numerous (90–95%), longest lived, and most widely distributed cells in bone tissue. Osteocytes are terminally differentiated osteoblasts that arise from MSCs [62,63]. Osteocytes, as mechanosensors of bone, sense mechanical signals and transmit them into biochemical signals to maintain bone homeostasis [69,70]. Osteocytes can generate signals to regulate bone-forming osteoblasts and bone-resorbing osteoclasts to renew bone [71]. How osteocytes sense the mechanical loading of bone is still a subject of ongoing research.

Recently, several studies demonstrated the expression of Piezo1 in osteocytes and its key role in mechanotransduction of osteocytes. Li et al. discovered that Piezo1 is upregulated by fluid shear force in MLO-Y4 osteocytes in in vitro research [32]. To verify the function of Piezo1 in osteocytes in vivo, they created mouse models that specifically deleted Piezo1 in osteocytes and osteoblasts [32]. The bones of Piezo1-knockout mice are small and weak. With +1200 με peak strain in leg bones, the bones of the unmodified mice increased in thickness after two weeks, whereas the bones lacking Piezo1 did not [32]. To understand the molecular mechanisms by which Piezo1 increases bone mass, Li et al. found that Piezo1 promotes Wnt1 (wingless-type MMTV integration site family, member 1) expression in osteocytes by activating YAP1 (Yes-associated transcriptional regulator 1) and TAZ (transcriptional coactivator with PDZ-binding motif) [32]. The Wnt1 signal pathway activated by Piezo1 in osteocytes, leads not only to increases in bone formation but also to decreases in bone resorption [32]. Recently, Sasaki et al. found that activation of Piezo1 activated by mechanical stretch in osteocytes can mediate phosphorylation of Akt (protein kinase B, encoding product of the retroviral Ann gene v-Akt), which downregulates the expression of sclerostin [67]. Since sclerostin could lead to decreased bone mass [72], Piezo1 in osteocytes inhibits the expression of sclerostin by activating the Akt signal pathway to promote bone formation.

Therefore, these data indicate that Piezo1 in osteocytes is involved in mechanotransduction by activating downstream Wnt1 signaling and Akt signaling.

### 5.2. Piezo1 and Bone Marrow Mesenchymal Stem Cells (BM-MSCs)

Bone marrow mesenchymal stem cells (BM-MSCs) are located in bone marrow and can self-renew [73]. In osteoblastogenesis, mesenchymal stem cells (MSCs) can differentiate into osteoblasts and eventually into osteocytes [74]. Therefore, MSCs play an important role in maintaining normal bone homeostasis [75,76]. Adipogenesis and osteoblastogenesis are two opposite directions of differentiation of mesenchymal stem cells. Adipogenesis-inducible factor inhibits osteoblastogenesis, while osteoblastogenesis-inducible factor blocks adipogenesis [77]. The specific direction of differentiation is precisely regulated by biological, physical, and chemical factors. Physical factors, such as mechanical strain, vibration, and hydrostatic pressure, are important factors in the osteogenesis of MSCs [76]. Sugimoto et al. established a cell culture chamber capable of controlling hydrostatic pressure, in which the increased expression of Piezo1 was detected and the osteogenic differentiation was enhanced in primary MSCs and MSC lines in in vitro research [33]. When MSCs were treated with Yoda1 in vitro, BMP2 (bone morphogenetic protein 2) expression was increased and promoted the differentiation to osteoblasts, which inhibited the differentiation to adipocytes [33]. The results of Sugimoto et al. suggest that mechanical stimulation of hydrostatic pressure induces Piezo1 to promote the differentiation of MSCs into osteoblasts by promoting the expression of BMP2 [33]. Because the above findings of Piezo1 in MSCs are from in vitro studies, further in vivo investigation is needed.

### 5.3. Piezo1 and Osteoblasts

Osteoblasts are mainly derived from MSCs in the inside and outside of periosteum and the matrix of bone marrow [78]. Mechanical loads associated with body weight, movement, and gravity normally help osteoblasts to build new bone tissue, which ensures that bone grows correctly and remains strong [79,80]. However, mechanical unloading of bone disrupts this process, leading to rapid bone loss [80]. Sun et al. found that Piezo1 is expressed in osteoblasts and helps osteoblasts respond to the mechanical shock of being poked by a microprobe in vitro [66]. Mice with Piezo1 specifically knocked out in osteoblasts failed to grow normally and were stunted in adulthood [66]. Furthermore, data on mice with hindlimb suspension in vivo and osteoblasts with a cell rotation system in vitro suggest that mechanical unloading can inhibit the expression of Piezo1, resulting in dysfunction of osteoblasts and bone formation [66]. Yan et al. silenced Piezo1 with small interfering RNA (siRNA) in the MC3T3-E1 osteoblasts [81]. Subsequent transwell cell migration experiments and cell scratch experiments showed that the number of Piezo1-siRNA cells migrating per well and the rate of scratch healing were significantly reduced, indicating that the Piezo1 gene silencing significantly inhibited the migration ability of MC3T3-E1 osteoblasts [81]. Meanwhile, Yoneda et al. found that Piezo1 activator Yoda1 triggers Ca^2+^ influx and promotes proliferation in MC3T3-E1 osteoblasts in vitro [82]. In vitro, MC3T3-E1 osteoblasts required Piezo1 to adapt to the external mechanical fluid shear stress, thereby inducing osteoblastic Runx2 (Runt-related transcription factor 2) gene expression, partly through the AKT/GSK-3β/β-catenin pathway [83]. Recently, Wang et al. have found a Piezo1-YAP1-collagen pathway in osteoblasts in vivo and in vitro [8]. More precisely, osteoclast differentiation is regulated by the expression of bone matrix proteins (collagen type II and IX), but these collagens are controlled by Piezo1 in osteoblasts via regulating nuclear translocation of YAP1 [8]. The Piezo1-YAP1-collagen pathway suggests that Piezo1 indirectly regulates bone resorption activity in osteoclasts, thereby affecting bone metabolism.

Interestingly, Zhou et al. indicated that while Piezo2 is dispensable for bone development, it shares redundant functions with Piezo1 in vivo [7]. Deficiency of Piezo1 and Piezo2 in osteoblasts results in more severe bone loss in mice than deficiency of Piezo1 [7]. In vitro, Piezo1 and Piezo2 convert mechanical signals (fluid shear stress and extracellular matrix stiffness) into intracellular Ca^2+^ signaling that activates calcineurin, which promotes concerted activation of NFATc1 (nuclear factor of activated T-cells, cytoplasmic 1), YAP1, and β-catenin transcription factors as well as NFAT/YAP1/β-catenin complex formation [7]. This process ultimately promotes osteoblast differentiation [7].

In summary, Piezo1 is a mechanical sensor in osteoblasts and plays a key role in mechanotransduction of osteoblasts. Piezo1 activates the Piezo1-YAP1-collagen pathway, activates the NFAT/YAP1/β-catenin transcription factor complex with Piezo2, activates the AKT/GSK-3β/β-catenin pathway, and reduces cell proliferation but increases its ability to migrate.

### 5.4. Piezo1 and Osteoclasts

Multinucleated osteoclasts are differentiated from cells of the myeloid lineage at various stages of maturity [84]. Osteoclasts are mainly responsible for initiating normal bone remodeling and mediating bone loss in pathologic conditions by increasing their resorptive activity [85]. Attaching to the old bone area and sensing surrounding mechanical environments, osteoclasts secrete acid and protease to digest the bone matrix and form a bone absorption cavity [86,87]. During bone remodeling, osteoclasts are regulated by osteoblasts and osteocytes to maintain bone homeostasis [71,88]. Sun et al. detected the expression of Piezo1 and cationic current induced by mechanical poking on the cell membrane in the pre-osteoclast cell line RAW264.7 [66]. To test whether Piezo1 affected the bone resorption of osteoclasts, Wang et al. deleted Piezo1 from mouse osteoclasts in vivo [8]. Consequently, bone resorption and bone mass in mice with Piezo1-deficiency in osteoclasts were basically unchanged compared with control mice. These findings suggest that Piezo1 has no role in osteoclasts, but whether Piezo2 has any role in osteoclasts is unknown.

### 5.5. Piezo1 and Chondrocytes

Chondrocytes, the cells in articular cartilage, regulate their metabolic activities in response to mechanical loading [65]. In diarthrodial joints, which allow a large degree of movement, the surfaces of the opposing bones are lined with hyaline cartilage, which reduces friction [89]. Chondrocytes are derived from MSCs [90]. Chondrocytes experience a complex mechanical environment and respond to changing mechanical loads in order to maintain cartilage homeostasis [91]. Chondrocyte mechanotransduction is not well understood, but recently, it was proposed that Piezo channels are of functional importance in chondrocyte mechanotransduction. Lee et al. detected robust expression of Piezo1 and Piezo2 in primary chondrocytes of mice, pigs, and humans [26]. Using a high-speed pressure clamp and elastomeric pillar arrays to apply distinct mechanical stimuli to primary mouse chondrocytes in vitro, Servin-Vences et al. found that Piezo1 contributes to currents activated by stretch of the membrane and deflection of cell-substrate contacts points [92]. In vitro, mechanical stress can cause Ca^2+^ to flow into chondrocytes through Piezo channels, resulting in cell apoptosis [26]. When Piezo1 or Piezo2 protein expression is inhibited by GsMTx4 in vitro, the Ca^2+^ transient effect activated by mechanical stress is eliminated [26]. In osteoarticular injury of chondrocytes in vitro, Li et al. suggested that Piezo1 participates in late apoptosis and that Piezo1 initiates the apoptosis process through the classic MAPK/ERK1/2 signaling pathway [68]. The next year, they also found that Piezo1 promotes chondrocyte apoptosis through the Caspase-12 (cysteine protease-12)-dependent pathway in chondrocytes derived from osteoarthritis in vitro [22]. In addition, a study showed that uridine can shut off Piezo1 in vitro, which ultimately protects chondrocytes from apoptosis by increasing cAMP (cyclic adenosine monophosphate) and subsequently inhibiting the expression of PLA_2_ (phospholipase A2), which may be closely related to p53-dependent apoptosis [93].

Mechanically activated ion channel TRPV4 (transient receptor potential vanilloid 4) is also of functional importance in chondrocyte mechanotransduction [94]. In vitro, TRPV4-mediated Ca^2+^ signaling played a central role in the response of chondrocytes to physiologic levels of strain, while Piezo2-mediated Ca^2+^ signaling played a central role in the response of chondrocytes to injurious levels of strain [95]. This result provides a possibility for therapeutically targeting Piezo2-mediated mechanotransduction for the treatment of OA that is induced by injurious and repetitive mechanical stimulation.

Piezo1 also plays an important role in the endochondral ossification in which chondrocytes are involved. Recently, by means of generating mice with Piezo1 deletion in chondrocytes, Hendrickx et al. found early-onset osteoporosis with multiple fractures in these mice [96]. This result shows that Piezo1 inactivation in growth-plate chondrocytes impairs trabecular bone formation.

These findings suggest that Piezo1 play a key role in the mechanotransduction and apoptosis of chondrocytes and in endochondral ossification.

Through the summary of Section 5, the functions and mechanism of Piezo1 in bone cells are shown in Table 2.

## 6. Piezo Channels and Bone Disease

Because Piezo channels play important roles in regulating bone physiology, recent studies demonstrate the involvement of Piezo channels in bone disease. Here, we mainly introduce the role of Piezo channels in osteoporosis (OP) and osteoarthritis (OA).

### 6.1. Osteoporosis (OP)

Osteoporosis (OP) is a progressive bone disease characterized by low bone mass and degeneration of bone microstructure, which leads to increased bone brittleness and an increased risk of fracture [76]. Many studies have shown that proper exercise or mechanical stimulation can prevent or treat OP [97,98,99].

Several groups have shown that mechanosensitive Piezo1 is a critical mediator in bone tissue that regulates bone loss. Li et al. demonstrated that Piezo1 plays a key role in maintaining bone homeostasis by regulating the perception of osteoblasts and/or osteocytes to mechanical loading in vivo and in vitro [32]. Activation of Piezo1 by Yoda1 mimics the promotion of Ca^2+^ influx effects of fluid shear stress on osteocytes in vitro. Corresponding to in vitro results, Yoda1 increases bone mass in mice [32]. Sun et al. constructed mice with knocked out Piezo1 in the osteoblastic lineage and further observed severe damage to bone structure and bone strength [66]. Consistent with this, Wang et al. constructed mice with knocked out Piezo1 from osteoblasts and found that the loss of Piezo1 in the osteoblasts led to severe OP [8]. Unexpectedly, Piezo1 additionally represents an essential osteogenic differentiation factor during endochondral ossification [96]. Mice with Piezo1 deletion in chondrocytes are found to exhibit early-onset osteoporosis with multiple fractures [96]. In human OP patients whose bones become weak with age, mRNA and protein expression levels of Piezo1 are significantly reduced [66]. Furthermore, the expression of Piezo1 was positively correlated with the expression of the marker genes of osteoblasts, including ALP (alkaline phosphatase), OCN (osteocalcin), and COL1A1 (collagen 1), in these human samples [66]. However, there are no correlations between the expression of Piezo1, osteoclast marker genes, and osteocyte marker genes [66]. These data suggest low expression of Piezo1 is correlated with defective osteoblast function and increasing bone loss [66].

Rolvien et al. proposed a model of the molecular pathways involved in disuse osteoporosis from mechanistic insights [100]. In response to unloading, inactivation of Piezo1 inhibits Wnt1 expression in osteocytes. Unloading leads to increased RANKL expression in osteocytes, promoting increased bone resorption. Unloading also leads to increased sclerostin in osteocytes, which inhibits Wnt/β-catenin and further inhibits bone formation of osteoblasts [100].

Polycystin-1 (PC1) and polycystin-2 (PC2) are a pair of conjugated mechanosensitive receptor channel complexes on the cell membrane that regulate bone mass [101,102,103,104,105,106,107]. Conditional deletion mice of PC1 in MSCs, osteoblasts, and osteocytes, respectively, have all showed significant reductions in bone mass due to decreased osteoblast-mediated bone formation in vivo [102,105,106]. Similarly, conditional deletion mice of PC2 in osteoblasts results in a reduction in bone mineral density, trabecular bone volume, and cortical thickness in vivo [107]. Xiao et al. have summarized that PC1, PC2, and TAZ, which responds to the flow shear force, act in concert to reciprocally promote osteoblastogenesis through co-activating Runx2 and co-repressing PPARγ (peroxisome proliferator-activated receptor γ) activities [108]. In addition, human GWAS (genome-wide association) studies also link PC2 with OP [109]. At present, triptolide has been found to be an agonist for PC2 and can restore Ca^2+^ signaling to attenuate overall cyst formation in kidney tubular epithelial cells in vitro [110]. Therefore, whether triptolide can be used to treat OP by activating PC2 is an urgent question to be verified.

These results suggest that mechanically activated ion channels, including Piezo1, PC1, and PC2, are closely related to the development of OP. Piezo1, PC1, and PC2 are novel therapeutic targets for OP, and their activator may be able to treat OP.

### 6.2. Osteoarthritis (OA)

Osteoarthritis (OA) is a painful and debilitating condition in synovial joints. It is characterized by progressive destruction of articular cartilage and chondrocyte apoptosis [64]. Under normal loading acting on normal physiology, chondrocytes undergo a complex mechanical environment and respond to changing mechanical loading to maintain cartilage homeostasis [64]. However, cartilage-damaging altered joint loading associated with obesity, knee malalignment, trauma, or joint instability, leads to maladaptive cellular mechanical responses and subsequent OA [64].

GsMTx4 is the inhibitor of many mechanosensitive ion channels [54]. The intra-articular injection of GsMTx4 significantly reduced the activation of dorsal horn nociceptive circuits and primary mechanical allodynia in OA mice [111]. Lee et al. revealed that the use of GsMTx4 can reduce the apoptosis rate of chondrocytes in vitro [26]. This phenomenon suggests that attenuation of mechanotransduction mediated by Piezo channels in damaged chondrocytes can reduce cartilage damage and post-traumatic OA [26]. Li et al. cultured human OA-derived chondrocytes and then applied static compression stimulation in vitro [22]. By detecting the expression levels of Piezo1 and apoptosis-related proteins caspase-12, Piezo1 and caspase-12 of OA-derived chondrocytes were found to be significantly upregulated under static compression stimulation [22]. These chondrocytes showed a tendency toward late apoptosis. In addition, GsMTx4 inhibited the expression of caspase-12 and late apoptosis in OA-derived chondrocytes [22]. Therefore, Piezo1 plays an important role in the apoptosis of human OA-derived chondrocytes through the caspase-12-dependent pathway in vitro [22].

Interleukin-1α (IL-1α) was found to upregulate Piezo1 in porcine chondrocytes in vitro, which resulted in a feed-forward pathomechanism whereby increased function of Piezo1 induced excess Ca^2+^ influx in response to mechanical deformation [112]. Elevated resting state Ca^2+^ in turn rarefied the F-actin cytoskeleton and amplified mechanically induced deformation microtrauma [112]. Increased Piezo1 expression depends on transcription factor CREBP1 which directly binds to the proximal Piezo1 gene promoter [112]. Thus, targeted inhibition of IL-1α- CREBP1-Piezo1 can inhibit the development of OA.

Transient receptor potential channel 4 (TRPV4) of chondrocytes could respond to mechanical stress and induce extracellular Ca^2+^ influx, thereby upregulating levels of fas-related proteins and caspase-3, caspase-6, caspase-7, and caspase-8, triggering chondrocyte apoptosis in a rat OA model [113]. However, inhibition of TRPV4 during dynamic loading prevented expression of anabolic and anticatabolic genes and inhibited the loading-induced enhancement of matrix accumulation and mechanical properties [94]. Furthermore, TRPV4 GSK1016790A enhanced anabolic and anticatabolic gene expression and increased matrix biosynthesis and mechanical properties [94]. Based on the improvement/deterioration effects of TRPV4 in chondrocytes, the issue of whether mechanosensitive ion channel-inhibitor GsMTx4 contributes to the treatment of OA is an urgent problem to be solved.

In conclusion, targeting mechanosensitive ion channels, such as Piezo channels and TRPV4, is the direction of future research for OA treatment. Moreover, based on the finding that activation of mechanosensitive ion channels promotes chondrocyte apoptosis, blockers of mechanosensitive ion channels may be useful in the treatment of OA.

Through the summary of Section 6, the role of Piezo1 in bone disease and associated mechanism are shown in Table 3.

## 7. Conclusions and Perspective

Bone, as the main mechanical support structure of the body, is continuously exposed to both external and internal mechanical stimuli, which play critical roles in bone physiology and pathology [101,102]. Thus, mechanotransduction has a key role in bone development, physiology, and disease conditions. Therefore, uncovering the mechanism of bone mechanotransduction is important for understanding both the maintenance of bone physiology and the development of bone disease as well as for providing therapeutic targets or methods for treating bone disease. Recent studies demonstrate the role of Piezo channels, especially Piezo1, in bone mechanotransduction and suggest its function in bone disease. Thus, the role of Piezo channels in bone mechanotransduction has become a research focus. Here, we review the recent advances of Piezo channels’ function in bone cells and in bone diseases, focusing on OP and OA.

Current research indicates that the Piezo channels, functioning as mechanotransducer, have a critical role in bone development and physiology, and are involved in bone diseases, including high-profile OP and OA. Piezo channels show a key role in maintaining bone homeostasis by mediating the mechanotransduction of bone cells. In osteocytes, Piezo1 promotes Wnt1 expression by activating YAP1 and TAZ. Since sclerostin could lead to decreased bone mass, Piezo1 in osteocytes inhibits expression of sclerostin by activating the Akt signal pathway. Both Wnt1 signing and Akt signing activated by Piezo1 in osteocytes promote bone formation of osteoblasts. The activation of Piezo1 in MSCs by mechanical loading promotes osteoblastogenesis differentiation of MSCs by upregulating BMP2. This implies that activation of Piezo1 in MSCs can increase the number of osteoblasts and can thus promote bone formation. In osteoblasts, key cells responsible for bone formation, the loss of Piezo1 leads to severe bone loss. In terms of specific mechanisms, the inactivation of Piezo1 in osteoblasts inhibits osteoblast differentiation by the AKT/GSK-3β/β-catenin pathway. Interestingly, deficiency of Piezo1 and Piezo2 in osteoblast results in more severe bone loss in mice than deficiency of Piezo1. In osteoblasts, Piezo1 can promote formation of the NFAT/YAP1/β-catenin transcription factor complex with Piezo2, resulting in increased bone formation. Piezo1 in osteoblasts not only promotes the bone formation of osteoblasts but also indirectly inhibits bone resorption of osteoclasts by the Piezo1-YAP1-collagen pathway. The involvement of chondrocytes in endochondral ossification is also closely related to bone formation. Mice with Piezo1 deletion in chondrocytes have multiple fractures. Piezo1 inactivation in chondrocytes of the growth plate impairs trabecular bone formation. Interestingly, mice with conditional deletion of Piezo1 in osteoclasts show no differences with normal mice, suggesting that Piezo1 may not function in osteoclasts. All these findings demonstrate the critical role of Piezo channels, especially Piezo1, in bone physiology. Thus, it is not surprising to find that Piezo channels are involved in the development of bone diseases. Both the clinical phenotypes and experimental mouse models demonstrate the key role of Piezo channels in bone diseases, including OP and OA. In OP patients, the low expression of Piezo1 is correlated with defective osteoblast function and increasing bone loss. Moreover, mice with conditional deletion of Piezo1 and/or Piezo2 in osteocytes, osteoblasts, and chondrocytes, respectively, develop OP. Piezo is not only associated with the development of OP, but it is also associated with deterioration in OA. In human OA-derived chondrocytes, upregulated Piezo1 promotes apoptosis of chondrocytes by activating caspase-12 signaling. These findings suggest that Piezo channels may be a potential target for the treatment of OP and OA. Activators of Piezo channels may hold promise for treating OP, and inhibitors of Piezo channels may alleviate OA. Activation of Piezo1 by Yoda1 can mimic the signaling cascade effects of mechanical loads on bone cells in vitro. Corresponding to in vitro results, Yoda1 increases bone mass in mice. Piezo channel-inhibitor GsMTx4 can reduce the apoptosis rate of chondrocytes in vitro.

The current findings on the role of Piezo channels in bone are very interesting and encouraging. Nevertheless, various questions also need to be addressed for better understanding of the function of Piezo channels in bone and for improving the treatment of bone diseases. Because the function of Piezo1 in bone has been studied more, what is the function of Piezo2 in bone? Is there an interaction or compensation action between Piezo1 and Piezo2 in different types of bone cells? Is there a leading role of Piezo1 or Piezo2 in bone? What is the underlying mechanism of Piezo channels involved in bone diseases such as OP and OA? Is there a specific role of Piezo1 or Piezo2 in specific bone disease? Given the broad expression and biological roles of Piezo1 in various tissues and cell types, how should Piezo1 be specifically targeted to treat bone disease? For answering these questions, some experimental mouse models or cell models with genetic mutations (knockout or knockin) of Piezo1, Piezo2, or Piezo1/Piezo2 should be generated in specific bone cell types and in combination with bone disease models to study the specific role and mechanism of Piezo1 and Piezo2 in normal bone physiology and bone disease. In addition, uncovering the function of the key structure domain of Piezo1 and Piezo2 will be useful for understanding the mechanotransduction mechanism of Piezo1 and Piezo2. Furthermore, it will be helpful to identify specific activators and inhibitors of Piezo1 and Piezo2 based on their structure, both for studying their physiopathological role and for investigating therapeutic methods. Answering these questions will provide deep understanding of bone physiology and pathology and will make Piezo channels a novel target for bone disease therapy.

## Figures and Tables

**Table 1:** The similarities and differences between Piezo1 and Piezo2.

Items	Piezo1	Piezo2	Reference
Gene	Fam38A	Fam38B	[17]
Chromosomal localization	Human chromosome 16	Human chromosome 18	NCBI database (https://www.ncbi.nlm.nih.gov/gene/9780 (accessed on 06 June 2021); https://www.ncbi.nlm.nih.gov/gene/63895 (accessed on 06 June 2021))
Amino acid size in humans	2521 amino acids	2752 amino acids	[34]
Amino acid size in mice	2547 amino acids	2822 amino acids	[35,36]
Structure	A homotrimer structure resembling a three-bladed propeller	A homotrimer structure resembling a three-bladed propeller	[36,37]
Transmembrane pore characteristics	Dilated	Closed	[36]
Tissue distribution	Widely distributed in skin, bladder, kidney, lung, endothelial cells, erythrocytes, periodontal ligament cells, trigeminal sensory neurons, dorsal root ganglion, etc.	Trigeminal sensory neurons, dorsal root ganglion, Merkel cells, and somatic neuron cells, etc.	[19,27,28,34,38]
Function	Involving in mechanotransduction in a variety of cells	Sensing slight touch and proprioception	[23,25,26,27,28,29,30,31]
Activator	Yoda1, Jedi1/2	Not found yet	[39,40]
Inhibitor	Ruthenium red, gadolinium, streptomycin, and GsMTx4	Ruthenium red, gadolinium, streptomycin, GsMTx4, and FM1-43	[17,19,41,42,43]

**Table 2:** Functions and mechanism of Piezo1 in bone cells.

Cell Type	Piezo1’s Functions	Mechanism	Reference
Osteocytes	Promotes bone formation regulated by osteocytes	Activates downstream Wnt1 signaling and Akt signaling	[32,67]
Mesenchymal stem cells	Promotes the differentiation of MSC into osteoblasts	Upregulates BMP2	[33]
Osteoblasts	Promotes bone formation by osteoblast; reduces cell proliferation but increases its migration ability; indirectly inhibits bone resorption of osteoclasts	Activates Piezo1-YAP1-collagen pathway; activates NFAT/YAP1/β-catenin transcription factor complex with Piezo2; activates AKT/GSK-3β/β-catenin pathway	[7,8,66,81,82,83]
Osteoclasts	No	No	[8]
Chondrocytes	Promotes chondrocyte apoptosis; promotes endochondral ossification in which chondrocytes are involved	Activates Ca^2+^ transient; activates MAPK/ERK1/2 pathway; activates caspase-12-dependent pathway;	[22,26,68,96]

**Table 3:** The role of Piezo1 in bone disease and associated mechanism.

Cell Type	Piezo1’s Functions	Mechanism	Reference
Osteoporosis	Downregulation in osteocyte and/or osteoblast; promotion of bone resorption leading to imbalance among bone formation and bone resorption under mechanical unloading	Unclear	[8,32,66]
Osteoarthritis	Upregulation in damaged chondrocytes; promotion of apoptosis of chondrocytes; rarefication of the F-actin cytoskeleton and amplification of mechanically induced deformation microtrauma	Activates caspase-12 signaling; promotes excessive Ca^2+^ influx, which in turn rarefies the F-actin cytoskeleton	[22,26,112]
